# Efficacy and safety of pyrotinib in advanced lung adenocarcinoma with *HER2* mutations: a multicenter, single-arm, phase II trial

**DOI:** 10.1186/s12916-022-02245-z

**Published:** 2022-02-01

**Authors:** Zhengbo Song, Yuping Li, Shiqing Chen, Shenpeng Ying, Shuguang Xu, Jianjin Huang, Dan Wu, Dongqing Lv, Ting Bei, Shuxun Liu, Xiaoping Huang, Congying Xie, Xiaoyu Wu, Jianfei Fu, Feng Hua, Wenxian Wang, Chunwei Xu, Chan Gao, Shangli Cai, Shun Lu, Yiping Zhang

**Affiliations:** 1 Department of Clinical Trial, Zhejiang Cancer Hospital, Hangzhou, 310022 China; 2 Department of Respiratory Diseases, The First Affiliated Hospital of Wenzhou Medical University, Wenzhou, 325000 China; 3 The Medical Department, 3D Medicines Inc., Shanghai, 201114 China; 4 Department of Radiotherapy, Taizhou Central Hospital, Affiliated Hospital of Taizhou University, Taizhou, 318000 China; 5 Department of Respiratory Disease, Ningbo Medical Center, Lihuili Eastern Hospital, Ningbo, 315001 China; 6 Department of Medical Oncology, the Second Affiliated Hospital, School of Medicine, Zhejiang University, Hangzhou, 310009 China; 7 Department of Thoracic Surgery, Cixi People Hospital, Ningbo, 315300 China; 8 Department of Respiratory Disease, Taizhou Hospital, Taizhou, 317000 China; 9 Department of Medical Oncology, Taizhou Cancer Hospital, Hangzhou, 317500 China; 10 Department of Respiratory Diseases, the Affiliated Ningbo No. 1 Hospital, School of Medicine, Ningbo University, Ningbo, 315001 China; 11 Department of Radiation and Medical Oncology, The Second Affiliated Hospital of Wenzhou Medical University, Wenzhou, 325000 China; 12 Department of Respiratory Diseases, Guangfu Hospital, Jinhua, 321000 China; 13 Department of Medical Oncology, Jinhua Central Hospital, Jinhua, 321000 China; 14 Department of Respiratory Diseases, Huzhou Central Hospital, Huzhou, 313003 China; 15 Department of Chemotherapy, Zhejiang Cancer Hospital, Hangzhou, 310022 China; 16 Department of Medical Oncology, Zhejiang Cancer Hospital, 1 East Banshan Road, Hangzhou, 310022 China; 17 Shanghai Lung Cancer Center, Shanghai Chest Hospital, Shanghai Jiao Tong University, Shanghai, 20030 China

**Keywords:** *HER2* mutations, Non-small cell lung cancer, Pyrotinib, Efficacy, Resistance mechanism

## Abstract

**Background:**

There is currently a lack of effective treatments for non-small cell lung cancer (NSCLC) patients harboring *HER2* mutations. We examined the efficacy and safety of, and potential resistance mechanism to, pyrotinib, a pan-HER inhibitor, in advanced NSCLC carrying *HER2* mutations.

**Methods:**

In this multicenter, single-arm, phase II trial, stage IIIB-IV NSCLC patients harboring *HER2* mutations, as determined using next-generation sequencing, were enrolled and treated with pyrotinib at a dose of 400 mg/day. The primary endpoint was 6-month progression-free survival (PFS) rate, and secondary endpoints were objective response rate (ORR), PFS, overall survival (OS), disease control rate (DCR), and safety. The impact of different *HER2* mutation types on sensitivity to pyrotinib and the potential of utilizing mutational profile derived from circulating tumor DNA (ctDNA) to predict disease progression were also explored.

**Results:**

Seventy-eight patients were enrolled for efficacy and safety analysis. The 6-month PFS rate was 49.5% (95% confidence interval [CI], 39.2–60.8). Pyrotinib produced an ORR of 19.2% (95% CI, 11.2–30.0), with median PFS of 5.6 months (95% CI, 2.8–8.4), and median OS of 10.5 months (95% CI, 8.7–12.3). The median duration of response was 9.9 months (95% CI, 6.2–13.6). All treatment-related adverse events (TRAEs) were grade 1–3 (all, 91.0%; grade 3, 20.5%), and the most common TRAE was diarrhea (all, 85.9%; grade 3, 16.7%). Patients with exon 20 and non-exon 20 *HER2* mutations had ORRs of 17.7% and 25.0%, respectively. Brain metastases at baseline and prior exposure to afatinib were not associated with ORR, PFS, or OS. Loss of *HER2* mutations and appearance of amplification in *HER2* and *EGFR* were detected upon disease progression.

**Conclusions:**

Pyrotinib exhibited promising efficacy and acceptable safety in NSCLC patients carrying exon 20 and non-exon 20 *HER2* mutations and is worth further investigation.

**Trial registration:**

Chinese Clinical Trial Registry Identifier: ChiCTR1800020262

**Supplementary Information:**

The online version contains supplementary material available at 10.1186/s12916-022-02245-z.

## Background

*HER2*-mutated non-small cell lung cancer (NSCLC) can only obtain limited clinical benefit from targeted therapies such as pan-HER tyrosine kinase inhibitors (TKIs) or TKIs targeting EGFR/HER1 or HER2 [1–3]. Although ado-trastuzumab emtansine (T-DM1) and fam-trastuzumab deruxtecan-nxki (T-DXd) are recommended as treatment options for advanced *HER2*-mutant NSCLC patients by the National Comprehensive Cancer Network (NCCN) guidelines based on ORRs of 44% (*N* = 18) and 72.7% (*N* = 11), respectively in advanced *HER2*-mutant lung adenocarcinomas, these two drugs have not been approved yet for treating this subset of patients [4, 5]. Chemotherapy remains the current standard-of-care for *HER2*-mutated NSCLC; however, it typically yields an ORR of 10–43.5% (1st-line, 43.5%; 2nd-line, 10%) and a PFS of 4.3-6 months (1st-line, 6 months; 2nd-line, 4.3 months) [6, 7]. Therefore, there exists an unmet need for effective HER2-targeting therapies to improve patients’ outcomes. Multiple NSCLC trials are ongoing to evaluate other novel TKIs, including tarloxotinib (NCT03805841), TAK-788 (NCT02716116), and poziotinib (NCT03318939; NCT04044170) [8].

Pyrotinib is an oral, irreversible pan-HER TKI, which has been adopted as the combination partner of capecitabine for treating advanced *HER2*- positive breast cancer in China [9]. In patient-derived lung cancer xenograft mouse models harboring *HER2* exon 20 insertions, pyrotinib demonstrated stronger antitumor activities than T-DM1 or afatinib [10]. In a phase II study (*N* = 60) conducted by Zhou C et al., chemotherapy-treated NSCLC patients with *HER2* mutations within exon 20 and 19 achieved an ORR of 30% upon pyrotinib, with mPFS of 6.9 months and median overall survival (mOS) of 14.4 months [11]. Evidence regarding efficacy and safety of pyrotinib remains to be confirmed in larger sample sizes, particularly in patients with *HER2* mutations outside of exon 20. Moreover, the underlying mechanism of resistance to pyrotinib and its efficacy in patients who had brain metastases and prior exposure to anti-HER2 therapy has not been well elucidated.

The aim of this study was to evaluate the efficacy and safety of pyrotinib in advanced NSCLC patients harboring *HER2* mutations. The impact of different *HER2* mutation types on sensitivity to pyrotinib, the association between baseline characteristics and response, and the potential of utilizing mutational profile information derived from circulating tumor DNA (ctDNA) to predict disease progression were also explored.

## Methods

### Patients

Patients were recruited at 11 Chinese hospitals from December, 2018 until April, 2020. Patients were enrolled if they were 18 years or older and had histocytologically confirmed unresectable stage IIIB or IV NSCLC, *HER2* mutations as determined using next-generation sequencing (NGS), an Eastern Cooperative Oncology Group (ECOG) performance status (PS) of 0-2, and at least one radiographically measurable lesion per Response Evaluation Criteria in Solid Tumors (RICIST) version 1.1 [12]. Exclusion criteria included having had undergone surgery, chemotherapy, or radiotherapy for NSCLC within 4 week before the study treatment. Written informed consent was provided by each patient before the onset of any trial-related treatment. The study protocol was approved by each site’s institutional review board in accordance with the Declaration of Helsinki and Good Clinical Practice guidelines.

### Study design and treatment

This is a multi-center, single-arm, phase II trial (Clinical trial registration: ChiCTR1800020262). Pyrotinib was administrated orally at 400 mg/day within 0.5 h after breakfast until intolerable toxicity, disease progression, or discontinuation at the patient’s request. In case of intolerable toxicity, the dose of pyrotinib was reduced to 320 mg daily. Depending on sample availability, biopsy tissue sample or blood sample was obtained from each patient at baseline, followed by NGS analysis. Under patients’ consents, blood samples were also collected from some patients upon disease progression for NGS analysis.

### Outcome assessment

The primary end point was 6-month PFS rate, which was defined as the proportion of PFS at 6 months after the first dose of pyrotinib. Secondary endpoints included safety, ORR (the frequency of patients who have had obtained partial response [PR] or complete response [CR] at two consecutive evaluations at least 4 weeks apart), PFS (the time between the first dose of pyrotinib and disease progression or death due to any reason), OS (the time between the first dose of pyrotinib and death due to any reason), and disease control rate (DCR, the frequency of patients who have had achieved a stable disease or PR or CR for ≥ 6 weeks before disease progression). Radiological assessment was conducted every six weeks in the first year, and every 9 weeks thereafter. Adverse events were assessed according to the National Cancer Institute Common Terminology Criteria for Adverse Events version 4.0. Upon disease progression, patients were followed up every 3 months until death. Exploratory endpoints included the association between different *HER2* mutation types and ORR, PFS, OS, or DCR and the feasibility of using ctDNA to monitor disease progression.

### Next-generation sequencing

Baseline tissue or blood samples were subjected to NGS-based molecular profiling to identify gene aberrations including alterations in the driver genes (*EGFR*, *ALK*, *ROS1*, *MET*, *BRAF*, *RET*, *HER2*, and *KRAS*) recommended by NCCN guidelines for NSCLC, while blood samples obtained from patients at disease progression were analyzed using a panel spanning 150 cancer-related genes at 3D Medicines, Inc., a clinical laboratory accredited by the College of American Pathologists (CAP) and certified by the Clinical Laboratory Improvement Amendments (CLIA) laboratory (Additional file 1: Supplementary Method for NG S[13, 14], Additional file 2: Table S1).

### Statistical analysis

According to previous study [15, 16], the 6-month progression-free rate of chemotherapy is hypothesized to be 30%, then 67 patients would provide 80% power to detect a 6-month progression-free rate of 45% at 5% alpha level. A total of 75 patients would need to be enrolled with the consideration of a dropout rate of 10%.

All statistical analyses were performed using the SPSS statistical software (version 20.0) and GraphPad prism (version 7). PFS and OS were estimated using Kaplan-Meier curves, with *P* value determined by a log-rank test. The difference in ORR and DCR between different groups were analyzed using the Fisher’s exact test. Cox regression was applied for calculating hazard ratio (HR) and 95% confidence intervals (CIs). A two-tailed *P* < 0.05 was defined as statistically significant.

## Results

### Patients

**Fig. 1:**
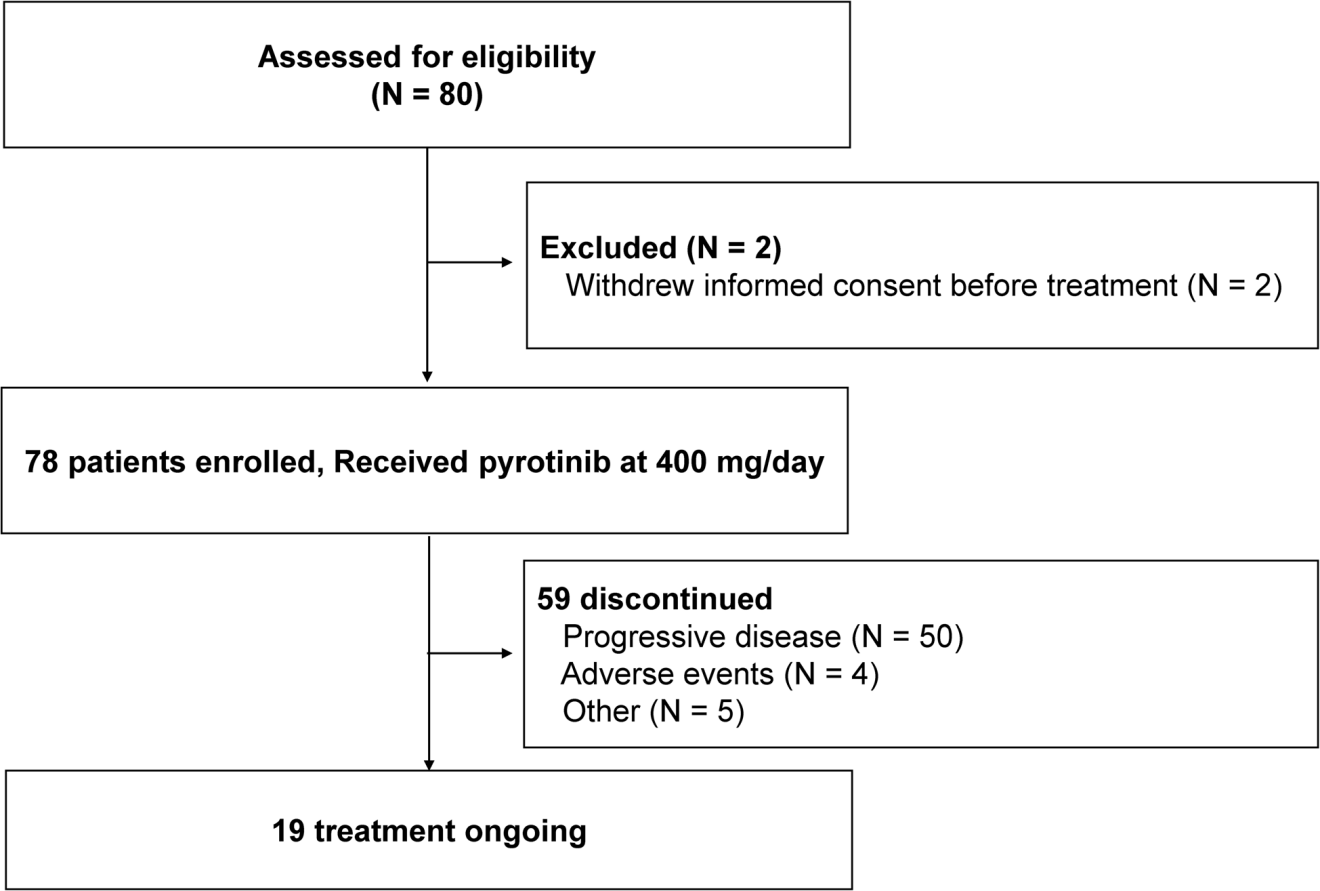
Study flow. We screened 80 patients and two patients were excluded due to withdrew informed consent before treatment. Hence the intention-to-treat population consisted of 78 patients, in which the efficacy and safety analyses were performed. As of December 2020, fifty-nine patients discontinued the study treatment for disease progression and intolerable adverse effect. The remaining 19 patients continued to receive pyrotinib

Between December, 2018 and April, 2020, 80 patients with *HER2* mutations were screened for eligibility. Two patients were excluded for withdrawing informed consents before study treatment; hence, a total of 78 patients were enrolled in this study and were included in the efficacy and safety analyses (Fig. 1). As data cut-off (December 30, 2020), the median duration of follow-up time was 10.5 months (range, 1.0–21.4 months). A total of 19 patients were still on treatment and 59 patients discontinued treatment, among which 50 for disease progression, 4 for intolerable adverse events, and the rest for other reasons.

Baseline characteristics were summarized in Table 1. The median age of the 78 patients was 62 years (range, 31–85 years). All patients had stage IV adenocarcinoma and 20 (25.6%) had brain metastases. Seven patients (9.0%) had an ECOG PS of 2 and the rest were 0–1. Most patients were non-smokers (65.4%). Twenty-one patients had a prior exposure to afatinib (first-line, *N* = 3; second-line or higher, *N* = 18). The majority of the patients received pyrotinib in the second-line or higher (first-line, 29.5%; second-line or higher, 70.5%). Among the enrolled patients, 62 carried *HER2* exon 20 mutations (79.5%) while the other 16 patients (20.5%) harbored mutations outside of exon 20. Of the 62 patients carrying exon 20 mutations, 42 and 11 patients had Y772_A775dup and G776delinsVC, respectively, and 9 carried other types of exon 20 mutations. Among the 78 patients, two patients harbored ≥ two *HER2* mutations. A total of 81 *HER2* mutations were detected at baseline, 73 fell in the kinase domain, three were in the transmembrane domain (TMD), three in extracellular domain, and the other two in other region of the coding region (Additional file 2: Fig. S1). *HER2* mutation types identified at baseline were summarized in Additional file 2: Table S2.

**Table 1:** Baseline characteristics

Characteristic		
Age, years		
	Median (range)	62 (31–85)
Sex, *n* (%)		
	Male	37 (47.4)
	Female	41 (52.6)
ECOG performance status, *n* (%)		
	0	15 (19.2)
	1	56 (71.8)
	2	7 (9.0)
Histology, *n* (%)		
	Adenocarcinoma	78 (100)
Stage, *n* (%)		
	IV	78 (100)
Brain metastases, *n* (%)		
	No	58 (74.4)
	Yes	20 (25.6)
Smoking status, *n* (%)		
	Former	22 (28.2)
	Never	51 (65.4)
	Unknown	5 (6.4)
*EGFR* mutation status, *n* (%)		
	Positive	6 (7.7)
	Negative	72 (92.3)
*ALK* fusion status, *n* (%)		
	Positive	0
	Negative	78 (100)
Pyrotinib treatment line, *n* (%)		
	1	23 (29.5)
	2	15 (19.2)
	≥ 3	40 (51.3)
Previous afatinib therapy		
	Yes	21 (26.9)
	No	57 (73.1)
*HER2* mutation, *n* (%)		
	Exon 20 mutation	62 (79.5)
	Non-exon 20 mutation	16 (20.5)

*ECOG* Eastern Cooperative Oncology Group

### Efficacy

**Fig. 2:**
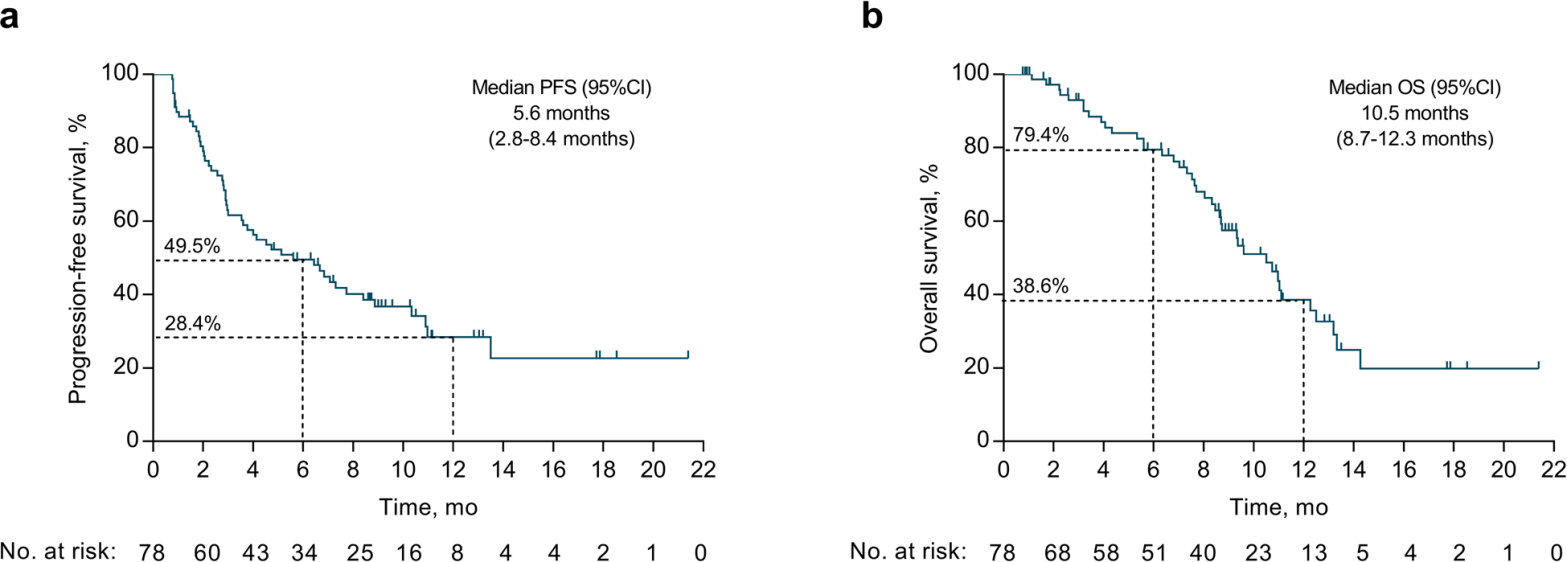
Kaplan-Meier survival curves of PFS and OS in pyrotinib treated NSCLC patients. PFS, progression-free survival; OS, overall survival; 95%CI, 95% confidence interval

**Fig. 3:**
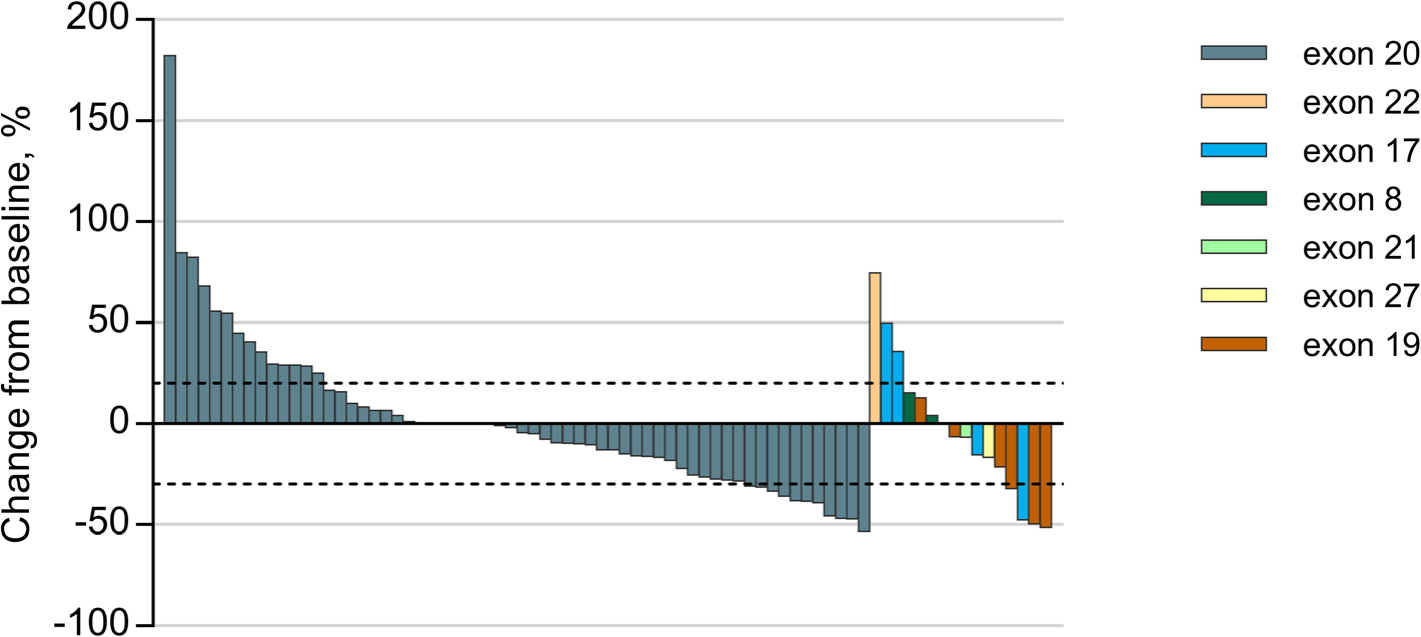
Tumor regression from baseline in primary lesions. Different colors demote mutations in different exons

As of December, 2020, the median duration of drug exposure was 5.6 months. A total of 50 PFS events and 40 deaths had occurred. The 6-month PFS rate was 49.5% (95% CI, 39.2–60.8%, Fig. 2). The 12-month PFS and OS rates were 28.4% and 38.6%, respectively. The mPFS and mOS were 5.6 months (95% CI, 2.8–8.4 months) and 10.5 months (95% CI, 8.7–12.3 months), respectively. Overall, 15 patients had a PR, for an ORR of 19.2% (15/78; 95% CI, 11.2–30.0%), including 11 patients with *HER2* mutations in exon 20, three in exon 19, and one in exon 17 (Table 2, Fig. 3). The median duration of response was 9.9 months (95% CI, 6.2–13.6 months), and the disease control rate was 74.4% (58 of 78; 95% CI, 63.2–83.6%). Of these 15 patients who responded to pyrotinib, seven received pyrotinib as the first-line treatment, two were previously treated with afatinib, and three had brain metastases. All these 15 patients had a PS score of 0–1.

**Table 2:** Clinical response to pyrotinib in NSCLC patients with *HER2* mutation

Variable	
Best response, *n* (%)	
Partial response	15 (19.2)
Stable disease	43 (55.1)
Progressive disease	20 (25.6)
Objective response rate, % (95% CI)	19.2 (11.2–30.0)
Disease control rate, % (95% CI)	74.4 (63.2–83.6)
Duration of response, median (95% CI)	9.9 (6.2–13.6)
Progression-free survival	
Events, *n* (%)	50 (64.1)
Median, months (95% CI)	5.6 (2.8–8.4)
Overall survival	
Events, *n* (%)	40 (51.3)
Median, months (95% CI)	10.5 (8.7–12.3)

*CI* confidence interval

When patients were stratified by baseline characteristics into comparison groups, we found that patients with a PS score of 2 displayed significantly worse OS than those with a PS score of 0–1 (mOS, 10.7 vs. 6.1 months; HR, 0.28; 95% CI, 0.11–0.75; *P* = 0.007) (Additional file 2: Fig. S2). The ORRs of patients who received pyrotinib in the first-line and secondary-line or higher were 30.4% and 14.5%, respectively (Additional file 2: Fig. S3). No significant difference in PFS or OS was observed among patients who received pyrotinib as the first-line treatment and those receiving pyrotinib in the secondary-line or higher setting (mPFS, 8.9 vs. 4.0 months; HR, 0.63; 95% CI, 0.33–1.18; *P* = 0.144; OS = 12.5 vs. 8.7 months; HR, 0.58; 95% CI, 0.28–1.18; *P* = 0.125) (Additional file 2: Fig. S4). The brain metastases at baseline and prior exposure to afatinib were not significantly associated with ORR, PFS, or OS (Additional file 2: Fig. S2-Fig. S4).

Upon dissection by *HER2* mutation types, the 62 patients harboring exon 20 mutations showed an ORR of 17.7% (95% CI, 9.2–29.5%) (Additional file 2: Fig. S3, Table S3). The ORRs for the patients harboring Y772_A775duplication, G776delinsVC, and other exon 20 mutations were 23.8% (95% CI, 12.1–39.5), 0.0% (95% CI, 0–28.5), and 11.1% (95% CI, 0.3–48.3), respectively. It was noteworthy that the ORR of the patients with non-exon 20 mutations reached 25.0%, which was comparable as seen in the patients harboring exon 20 mutations (25.0% vs. 17.7%; *P* = 0.495 ). Particularly, among the six patients with exon 19 mutations, three achieved PR, reaching an ORR to 50%. Of these three PR patients carrying exon 19 mutations, two were treated with pyrotinib as first-line treatment. In addition, among the three patients with TMD mutations, the two patients carrying V658E substitution showed PFS of 2.9–5.6 months and OS of 5.3–5.6 months, while the patient harboring I655V had PFS and OS of 0.8 and 1.13 months, respectively (data not shown). No significant differences in PFS or OS were observed between patients who had exon 20 and non-exon 20 mutations (Additional file 2: Fig. S5).

Patients harboring co-mutations in driver genes such as *EGFR*, *KRAS, BRAF*, and *ROS1* at baseline exhibited similar ORR (30.0% vs. 17.6%, *P* = 0.434) and mPFS (3.0 vs. 6.7 months; *P* = 0.294) to and a poorer mOS (6.8 vs. 11.0 months; *P* = 0.017) than their wild-type counterparts (Additional file 2: Fig. S3, Fig. S6). Patients with *EGFR* mutations had numerically inferior clinical outcomes than the *EGFR*-wild-type patients (ORR, 0 vs. 20.8%, *P* = 0.590; PFS, 3 vs. 6.4 months, *P* = 0.185). No difference was seen in ORR (19.4% vs. 16.7%; *P* = 1.000), PFS (5.4 vs. 14.0 months; *P* = 0.421), or OS (10.5 vs. NR months; *P* = 0.558) between patients without and with *HER2* copy number amplification (CNA) at baseline (Additional file 2: Fig. S3, Fig. S6).

### Safety

Treatment-related adverse events (TRAEs) of any grade occurred in 71 of 78 patients (91.0%), most of which were grade 1 or 2 (Table 3). Diarrhea was the most common TRAE (85.9%), followed by fatigue (57.7%), anemia (35.9%), dizziness (33.3%), decreased appetite (32.1%), hand-foot syndrome (32.1%), and nausea (32.1%). Sixteen patients suffered from grade 3 TRAEs (20.5%), including 13 diarrhea (16.7%), 2 anemia (2.6%), and 1 fatigue (1.3%). No grade 4 or higher TRAEs were observed. Four patients discontinued treatment as a result of TRAEs, two for grade 3 diarrhea, one for grade 2 fatigue, and one for grade 2 decreased appetite, nausea, and vomiting. Two patients had a dose reduction due to intolerable toxicity.

**Table 3:** Treatment-related adverse events

Adverse event	Pyrotinib (*n* = 78), *n* (%)			
	All Grades	Grade 1	Grade 2	Grade 3
Any	71 (91.0)	70 (89.7)	45 (57.7)	16 (20.5)
Occurring in ≥ 10% of patients				
Diarrhea	67 (85.9)	25 (32.1)	29 (37.2)	13 (16.7)
Fatigue	45 (57.7)	39 (50.0)	5 (6.4)	1 (1.3)
Anemia	28 (35.9)	18 (23.1)	8 (10.3)	2 (2.6)
Dizziness	26 (33.3)	25 (32.1)	1 (1.3)	
Decreased appetite	25 (32.1)	22 (28.2)	3 (3.8)	
Hand-foot syndrome	25 (32.1)	22 (28.2)	3 (3.8)	
Nausea	25 (32.1)	24 (30.8)	1 (1.3)	
WBC decreased	19 (24.4)	13 (16.7)	6 (7.7)	
Blood creatinine increased	19 (24.4)	19 (24.4)		
Cough	18 (23.1)	18 (23.1)		
ALT increased	17 (21.8)	17 (21.8)		
Vomiting	16 (20.5)	13 (16.7)	3 (3.8)	
Headache	16 (20.5)	16 (20.5)		
AST increased	15 (19.2)	15 (19.2)		
Hypokalemia	14 (17.9)	14 (17.9)		
Weight decreased	12 (15.4)	11 (14.1)	1 (1.3)	
Pain	12 (15.4)	12 (15.4)		
Hyponatremia	11 (14.1)	11 (14.1)		
Chest distress	10 (12.8)	9 (11.5)	1 (1.3)	

*ALT* alanine aminotransferase, *AST* aspartate aminotransferase, *WBC* white blood cell

*No grade 4 or higher adverse events occurred

### Feasibility of using ctDNA to monitor disease progression upon pyrotinib treatment

Of the 78 patients in the analysis cohort, twelve patients who acquired resistance to pyrotinib had blood samples available both at baseline and upon disease progression. These blood samples were subjected to NGS analysis to monitor disease progression. Concurrent *HER2* CNA and *EGFR* CNA, which were not presented at baseline blood samples, were detected from two patients upon PD, suggesting that co-occurrence of *HER2* CNA and *EGFR* CNA may have played a role in resistance to pyrotinib. One of these two patients’ representative CT images captured at baseline, best response, and PD are shown in Additional file 2: Fig. S7. Another four patients had a loss of *HER2* mutation upon PD, rending it rational to speculate that the loss of *HER2* mutations may confer resistance to pyrotinib. In addition, appearance of *EGFR* (p.E330K), *KRAS* (p.G12D), *MET* CNA, and *BRAF* CNA were also detected in three patients at PD (Additional file 2: Table S4). Since KRAS and BRAF are both downstream of HER2 in the RAS/RAF signaling pathway, our results suggested that gene alterations in the RAS/RAF pathway may serve as a potential mechanism of resistance to pyrotinib.

## Discussion

*HER2* mutations are rarely observed in NSCLC. There exists little evidence regarding effective treatment of NSCLC patients with *HER2* mutations, especially those with non-exon 20 mutations. Herein, we reported the effect of pyrotinib in 78 advanced lung adenocarcinoma patients harboring different types of *HER2* mutations. In the total population, pyrotinib produced 6-month PFS rate of 49.5%, mPFS of 5.6 months, mOS of 10.5 months, and ORR of 19.2%. In line with previous studies, the most common TRAE was diarrhea, and grade 3 diarrhea occurred in 16.7% of the patients. Among patients with *HER2* mutations in different exons, patients harboring non-exon 20 aberrations achieved comparable ORR than those with exon 20 mutations. Patients who had brain metastases and prior exposure to anti-HER therapy could benefit from pyrotinib. Moreover, loss of *HER2* mutations, appearance of *HER2* amplification, and aberrations in *EGFR, MET, KRAS*, and *BRAF* were detected upon disease progression, suggesting their potential roles in the resistance to pyrotinib.

Chemotherapy, the current standard treatment for advanced NSCLC patients with *HER2* mutations, typically elicits an ORR of 10% and an mPFS of 4.3 months in a second-line setting (6). TKIs targeting HER2 or pan-HER have been investigated for treating *HER2*-mutated lung cancer patients. However, afatinib, neratinib, and dacomitinib only elicited ORR of 7.7%, 3.8%, and 12% [1–3]. The ORRs upon T-DM1 and T-DXd treatment could reach up to 44% (8/18) and 72.7% (8/11), respectively [4, 5]. The mPFS of T-DM1-treated NSCLC patients as previously reported was 5.0 months, which was similar to that observed in the present study (5.0 vs. 5.6 months). Most recently, the results of the phase II study DESTINY-Lung trial were released in which T-DXd showed an ORR of 55% (50/91) and mPFS of 8.2 months in patients with previously treated NSCLC with *HER2* mutation [17]. Albeit encouraging anti-tumor activity, grade 4 and 5 TRAEs occurred upon T-DXd, whereas in our study, no grade 4 or 5 TRAEs were observed, suggesting that pyrotinib is safer than T-DXd [5, 17]. Poziotinib, another promising anti-HER2 TKI, has exhibited an ORR of 42% in *HER2*-mutated NSCLC patients (*N* = 12), causing grade 3 or 4 AEs in 66.7% of the patients [18].

Treatment of *HER2*-mutated NSCLC with pyrotinib has been previously reported. In phase II trials conducted by Wang Y et al. and Zhou C et al., treatment with pyrotinib was associated with ORRs of 53.3% and 30%, and mPFSs of 6.4 months and 6.9 months in cohorts of 15 and 60 *HER*-mutated advanced NSCLC patients [10, 11]. Both studies reported better efficacy than our observations (ORR, 19.2%; PFS, 5.6 months). This could have been explained by the fact that our study enrolled patients with a PS score of 2 (7/78, 9%) whereas Zhou C’s study only included patients with a PS score of 0–1. A higher percentage of patients in our cohort had brain metastases at baseline (25.6% vs. 20%) and more patients received pyrotinib in the third line or higher (51.3% vs. 41.6%) than in their study. In addition, patients who had prior exposure to HER2-targeted drugs were also included in our study. Of note, the duration of response in the present study was 9.9 months, which was longer than 6.9 months documented in Zhou C’s study.

The sensitivities to anti-HER2 TKIs in patients bearing different *HER2* mutations were also distinct. In patients with *HER2*-mutated NSCLC, the major *HER2* mutation type was exon 20 insertions, occurring in 1.5% of NSCLC and accounting for 90% of all NSCLC with *HER2* mutations [19–22]. Previous studies have been mainly focusing on these insertions. Two prospective studies investigating pyrotinib employed the ADx HER2 Mutation Detection Kit for *HER2* genotyping, which only allows for detection of exon 20 and 19 mutations [10, 11]. In our study, we utilized NGS to detect *HER2* mutations, which was capable of identifying mutations outside of exons 20 and 19. Indeed, patients carrying mutations outside of exon 20 were also able to benefit from pyrotinib. A numerically higher ORR was observed among patients carrying non-exon 20 mutations, especially those carrying exon 19 mutations. These observations were consistent with previous findings that *HER2* exon 20 insertions are less sensitive to currently available TKIs than mutations in other exons, potentially due to the structural difference of mutant in this exon from in others [19]. *HER2* exon 20 insertions primarily affected two structural regions: the αC- helix, comprising residues 770–774, and the loop region at residues 775–783 [20, 21, 23]. Structure-based comparison of behaviors between these variant types needs to be further studied.

Patients with *HER2* exon 20 mutation Y772_A775dup, the most common *HER2* mutation in NSCLC, failed to respond to afatinib and dacomitinib as reported [1, 24, 25]. Surprisingly, pyrotinib produced an ORR and a DCR of 23.8% and 78.6%, respectively, in 42 patients harboring Y772_A775dup in our study [24, 25]. Consistent with the results of Zhou C’s study, although none of the 11 patients carrying G776delinsVC achieved PR in our study, the DCR of this subset reached 63.6%, which was similar to that of the other mutation types [11]. Clinical efficacy regarding anti-HER2 TKIs has been poorly investigated in patients with *HER2* TMD mutations [26, 27]. In our study, three patients harbored *HER2* TMD, including two with V659E and one with I655V. The PFS and OS of the patients with V659E was 2.9–5.6 months and 5.3–5.6 months, respectively. The other patient bearing I655V, however, experienced PD three weeks after initiation of pyrotinib. Collectively, our results revealed variable efficacy of pyrotinib in NSCLC patients with different *HER2* mutations and warrant further validation in larger randomized clinical trials.

Another point to be noted was the monitoring of acquired resistance to pyrotinib by using blood sample profiling, highlighting the importance of liquid biopsy in this setting. In this study, we also explored potential resistance mechanisms underlying disease progression upon pyrotinib. *HER2* CNA was identified from two patients upon PD, consistent with a previous report that *HER2* CNA conferred resistance to anti-HER2 TKIs in *HER2*-mutated NSCLC patients [28]. Of note, *EGFR* CNA was also detected from these two patients upon PD, indicating the concurrent *HER2* CNA and *EGFR* CNA may engender resistance to pyrotinib. In another four PD patients, *HER2* mutation, which existed at baseline, was not detected from the blood sample at PD, rending it rational to speculate that the loss of *HER2* mutations may engender resistance to pyrotinib as well. In addition, *MET* CNA, *KRAS* (p.G12D), *BRAF* CNA, and *EGFR* (p.E330K) were also detected from patients at PD. *MET* CNA has been reported to be associated with resistance to anti-HER2 TKIs in *EGFR*-mutant NSCLC, *HER2*-amplified breast cancer, and *HER2*-mutated NSCLC [28–30]. Based on these results, we propose that strategies combining pyrotinib and EGFR TKI or other TKIs targeting the above alternations might be a potential treatment option to vanquish resistance or potentiate the antitumor activities in treating this subset of patients.

Indeed, Rolfo C et al. summarized a series of novel agents that has potential against *HER2*-mutated NSCLC [8]. Interestingly, the combinational treatment of a pan-HER inhibitor (neratinib) and T-DM1 or T-DXd induced a superior activity compared with T-DM1 alone [31]. Similarly, preclinical studies revealed that the novel pan-HER TKI poziotinib could up-regulate HER2 cell-surface expression and increase the activity of T-DM1 in tumors with *HER2*-mutation [32]. In addition, Bob T. Li et al. reported that the combination of T-DM1 and irreversible pan-HER inhibitors (neratinib or afatinib) could enhance the duration of the responses in HER2-altered lung cancers [31]. Pyrotinib is an irreversible pan-HER inhibitor, also presenting promising activity in *HER2*-mutated NSCLC as observed in our study. Part of data of this trial (ChiCTR1800020262) was published recently which has shown the efficacy of pyrotinib in NSCLC patients with *HER2* amplification (6-month PFS rate: 51.9%, ORR: 22.2%, mPFS: 6.3 months, mOS: 12.5 months) [33]. Therefore, a combination of T-DM1/T-DXd and pyrotinib may become a potentially effective therapy for these *HER2*-altered patients. These results indicate that combining T-DM1/T-DXd and anti-HER2 TKI might be a potential treatment option to increase antitumor activity or conquer resistance to targeted therapies. The above proposals are a ray of hope shining the future of patients with *HER2* alternations.

Despite being the largest prospective study investigating pyrotinib effects in NSCLC, our study is still limited by the small sample size due to the low prevalence of *HER2* mutations in NSCLC. Second, comparison with chemotherapy or other targeted therapies was not feasible due to a lack of control arm. The findings of the current study should be examined in larger randomized clinical trials.

## Conclusions

Pyrotinib exhibited promising efficacy and acceptable safety in treating NSCLC patients with both exon 20 and non-exon 20 *HER2* mutations.

## Supplementary Information

**Additional file 1: Supplementary Methods.**

**Additional file 2: Table S1-S4.**, **Figure S1-S7.** **Table S1.** List of genes in the 3DMed 150-gene panel. **Table S2.** *HER2* mutations identified at baseline. **Table S3.** Clinical response to pyrotinib according to different HER2 mutation types. **Table S4.** The detected molecular alterations at baseline and progression. **Fig. S1.** *HER2* mutational map at baseline. Green: receptor L domain; red: furin-like cysteine rich region; blue: growth factor receptor domain IV; yellow: protein tyrosine kinase. **Fig. S2.** Survival curves of pyrotinib treated *HER2*-mutated NSCLC patients according to baseline characteristics. (A, B) progression-free survival (PFS) and overall survival (OS) according to the ECOG performance status. PS represents ECOG performance score. (C, D) PFS and OS of pyrotinib treated patients with or without brain metastasis. mPFS, median progression-free survival; mOS, median overall survival; HR, hazard ratio; 95%CI, 95% confidence interval. **Fig. S3** Objective response rate in pre-specific subgroups. **Fig. S4** Survival curves of NSCLC patients treated with pyrotinib according to previous treatment. (A, B) progression-free survival (PFS) and overall survival (OS) according to the treatment lines of pyrotinib. (C, D) PFS and OS of patients according to the prior exposures to afatinib. mPFS, median progression-free survival; mOS, median overall survival; HR, hazard ratio; 95%CI, 95% confidence interval. **Fig. S5** Survival curves of pyrotinib treated NSCLC patients with different *HER2* mutation. mPFS, median progression-free survival; mOS, median overall survival; HR, hazard ratio; 95%CI, 95% confidence interval. **Fig. S6** Survival curves of pyrotinib treated *HER2*-mutated NSCLC patients according to molecular characteristics. (A, B) progression-free survival and overall survival according to *HER2* amplification. (C, D) PFS and OS of pyrotinib treated patients according to the occurrence of co-mutations in other driver genes. mPFS, median progression-free survival; mOS, median overall survival; HR, hazard ratio; 95%CI, 95% confidence interval. **Fig. S7** Pyrotinib resistance in a patient with *HER2* and *EGFR* amplification. CT scans were performed at baseline (2 weeks before starting pyrotinib), best response (2 months after starting pyrotinib) and disease progression (7 months after starting pyrotinib), respectively. Mm, millimeter.

## Data Availability

The datasets used and/or analyzed during the current study are available from the corresponding author upon reasonable request.

## Abbreviations

CAP: College of American Pathologists
CI: Confidence interval
CLIA: Clinical laboratory improvement amendments
CNA: Copy number amplification
CR: Complete response
ctDNA: Circulating tumor DNA
DCR: Disease control rate
ECOG: Eastern Cooperative Oncology Group
HER2: Human epidermal growth factor receptor 2
HR: Hazard ratio
NCCN: National comprehensive cancer network
NGS: Next-generation sequencing
NSCLC: Non-small cell lung cancer
ORR: Objective response rate
OS: Overall survival
PFS: Progression-free survival
PR: Partial response
PS: Performance status
RICIST: Response evaluation criteria in solid tumors
T-DM1: Ado-trastuzumab emtansine
T-DXd: Fam-trastuzumab deruxtecan-nxki
TKI: Tyrosine kinase inhibitor
TMD: Transmembrane domain
TRAE: Treatment-related adverse event
