# Supplementary material for: Efficacy and safety of pyrotinib in advanced lung adenocarcinoma with HER2 mutations: a multicenter, single-arm, phase II trial
Source: BMC Med. 2022 Feb 1;20:42. doi: 10.1186/s12916-022-02245-z (PMC8805254; doi:10.1186/s12916-022-02245-z)
Supplement: Supplementary file 1 — Additional file 1: Supplementary Methods. [file 12916_2022_2245_MOESM1_ESM.docx]

NGS and sequencing data analysis were performed as previously described [^1^](#_ENREF_1)^,^[^2^](#_ENREF_2). Briefly, cell-free DNA (cfDNA) was extracted using the QiAmp Circulating Nucleic Acid Kit (Qiagen). CfDNA libraries were constructed using the KAPA Hyper Prep Kit (KAPA Biosystems) and were individually barcoded with unique molecular identifiers (UMI), followed by probe-based hybridization. The captured libraries were subsequently loaded onto a NovaSeq 6000 platform (Illumina) for paired-end sequencing with a mean sequencing depth of 35000×. Sequencing data were aligned to the human genome hg19 and an in-house developed software was used to generate duplex consensus sequences based on dual UMI. To improve specificity, an in-house model for detecting loci specific variants was applied to improve specificity. Only missense, frameshift and non-frameshift indel mutations, and stop gain were kept. Gene rearrangements and copy number variations were also determined.
