# Supplementary material for: Efficacy and safety of pyrotinib in advanced lung adenocarcinoma with HER2 mutations: a multicenter, single-arm, phase II trial
Source: BMC Med. 2022 Feb 1;20:42. doi: 10.1186/s12916-022-02245-z (PMC8805254; doi:10.1186/s12916-022-02245-z)
Supplement: Supplementary file 2 — Additional file 2: Table S1-S4., Figure S1-S7. Table S1. List of genes in the 3DMed 150-gene panel. Table S2. HER2 mutations identified at baseline. Table S3. Clinical response to pyrotinib according to different HER2 mutation types. Table S4. The detected molecular alterations at baseline and progression. Fig. S1. HER2 mutational map at baseline. Green: receptor L domain; red: furin-like cysteine rich region; blue: growth factor receptor domain IV; yellow: protein tyrosine kinase. Fig. S2. Survival curves of pyrotinib treated HER2-mutated NSCLC patients according to baseline characteristics. (A, B) progression-free survival (PFS) and overall survival (OS) according to the ECOG performance status. PS represents ECOG performance score. (C, D) PFS and OS of pyrotinib treated patients with or without brain metastasis. mPFS, median progression-free survival; mOS, median overall survival; HR, hazard ratio; 95%CI, 95% confidence interval. Fig. S3 Objective response rate in pre-specific subgroups. Fig. S4 Survival curves of NSCLC patients treated with pyrotinib according to previous treatment. (A, B) progression-free survival (PFS) and overall survival (OS) according to the treatment lines of pyrotinib. (C, D) PFS and OS of patients according to the prior exposures to afatinib. mPFS, median progression-free survival; mOS, median overall survival; HR, hazard ratio; 95%CI, 95% confidence interval. Fig. S5 Survival curves of pyrotinib treated NSCLC patients with different HER2 mutation. mPFS, median progression-free survival; mOS, median overall survival; HR, hazard ratio; 95%CI, 95% confidence interval. Fig. S6 Survival curves of pyrotinib treated HER2-mutated NSCLC patients according to molecular characteristics. (A, B) progression-free survival and overall survival according to HER2 amplification. (C, D) PFS and OS of pyrotinib treated patients according to the occurrence of co-mutations in other driver genes. mPFS, median progression-free survival; mOS, median overall sur [file 12916_2022_2245_MOESM2_ESM.docx]

**Supplementary tables and figures**

**Table S1………………………………………………………………………………………………………………………………2**

**Table S2………………………………………………………………………………………………………………………………3**

**Table S3………………………………………………………………………………………………………………………………4**

**Table S4………………………………………………………………………………………………………………………………5**

**Figure S1…………………………………………………………………………………………………………………………….6**

**Figure S2…………………………………………………………………………………………………………………………….7**

**Figure S3…………………………………………………………………………………………………………………………….8**

**Figure S4…………………………………………………………………………………………………………………………….9**

**Figure S5……………………………………………………………………………………………………………………………10**

**Figure S6……………………………………………………………………………………………………………………………11**

**Figure S7……………………………………………………………………………………………………………………………12**

**Supplemental table 1. List of genes in the 3DMed 150-gene panel**

| ACVR2A | BRAF | CHEK1 | ERBB4 | GLI3 | KRAS | MYCN | PDGFRA | RIT1 | SRC |
| --- | --- | --- | --- | --- | --- | --- | --- | --- | --- |
| AKT1 | BRCA1 | CHEK2 | ERRFI1 | GNA11 | LRP1B | NF1 | PDGFRB | RNF43 | STK11 |
| AKT2 | BRCA2 | CREBBP | ESR1 | GNAQ | MAP2K1 | NFE2L2 | PIK3CA | ROS1 | TBX3 |
| ALK | BRIP1 | CRKL | EZH2 | GNAS | MAP2K2 | NKX2-1 | PIK3R1 | RUNX1T1 | TCF7L2 |
| APC | CBL | CTNNB1 | FAM135B | HNF1A | MAP2K4 | NOTCH1 | PREX2 | SETD2 | TERT |
| AR | CCND1 | CYP2C19 | FAT1 | HRAS | MAP3K1 | NOTCH2 | PTCH1 | SLIT2 | TGFBR2 |
| ARAF | CCND2 | CYP2D6 | FBXW7 | IDH1 | MCL1 | NOTCH3 | PTEN | SMAD2 | TP53 |
| ARID1A | CCNE1 | DDR2 | FGF19 | IDH2 | MET | NRAS | PTK2 | SMAD3 | TPMT |
| ARID2 | CD274 | DPYD | FGFR1 | IRS2 | MLH1 | NRG1 | PTPN11 | SMAD4 | TSC1 |
| ATM | CDH1 | EGFR | FGFR2 | JAK2 | MRE11 | NRG3 | RAD50 | SMARCA2 | TSC2 |
| ATR | CDK12 | EMSY | FGFR3 | JAK3 | MSH2 | NTRK1 | RAF1 | SMARCA4 | UGT1A1 |
| AXIN1 | CDK4 | EP300 | FLT1 | KDR | MSH6 | NTRK2 | RB1 | SMO | VEGFA |
| BARD1 | CDK6 | EPHB1 | FLT3 | KEAP1 | MTOR | NTRK3 | RBM10 | SOX2 | VHL |
| BCL2L11 | CDKN1B | ERBB2 | FLT4 | KIT | MYC | PALB2 | RET | SPEN | ZNF217 |
| BIRC5 | CDKN2A | ERBB3 | GATA3 | KMT2A | MYCL | PDCD1LG2 | RICTOR | SPTA1 | ZNF703 |

**Supplementary table 2. *HER2* mutations identified at baseline**

| *HER2* mutation type | exon | n | % |
| --- | --- | --- | --- |
| Y772_A775dup | 20 | 42 | 53.80 |
| G776delinsVC | 20 | 11 | 14.10 |
| G776delinsLC | 20 | 2 | 2.60 |
| G778_P780dup | 20 | 2 | 2.60 |
| G776A | 20 | 1 | 1.30 |
| G776delinsAVGA | 20 | 1 | 1.30 |
| G776delinsAVGS | 20 | 1 | 1.30 |
| G776delinsLV | 20 | 1 | 1.30 |
| G776S | 20 | 1 | 1.30 |
| G778_S779insAVG | 20 | 1 | 1.30 |
| V777_G778ins E | 20 | 1 | 1.30 |
| Y803F | 20 | 1 | 1.30 |
| L755P | 19 | 4 | 5.10 |
| S310F | 8 | 2 | 2.60 |
| V659E | 17 | 2 | 2.60 |
| S310Y | 8 | 1 | 1.30 |
| I655V | 17 | 1 | 1.30 |
| L674F | 17 | 1 | 1.30 |
| D769Y | 19 | 1 | 1.30 |
| L755S | 19 | 1 | 1.30 |
| H878Y | 21 | 1 | 1.30 |
| R896G | 22 | 1 | 1.30 |
| E1195G | 27 | 1 | 1.30 |

**Supplementary table 3. Clinical response to pyrotinib according to different *HER2* mutation types**

|  | exon 20 mutation | | | | other mutation |
| --- | --- | --- | --- | --- | --- |
|  | all exon 20 mutation | Y772_A775dup | G776delinsVC | other exon 20 mutation |  |
| Total, n | 62 | 42 | 11 | 9 | 16 |
| PR, n | 11 | 10 | 0 | 1 | 4 |
| SD, n | 35 | 23 | 7 | 5 | 8 |
| PD, n | 16 | 9 | 4 | 3 | 4 |
| ORR, % (95%CI) | 17.7 (9.2-29.5) | 23.8 (12.1-39.5) | 0 (0-28.5) | 11.1 (0.3-48.3) | 25 (7.3-52.4) |
| DCR, % (95%CI) | 74.2 (61.5-84.5) | 78.6 (63.2-89.7) | 63.6 (30.8-89.1) | 66.7 (29.9-92.5) | 75 (47.6-92.7) |

PR, partial response; SD, stable disease; PD, progressive disease; ORR, objective response rate; DCR, disease control rate; CI, confidence interval.

**Supplementary table 4. The detected molecular alterations at baseline and progression**

| Patient | Baseline | Progression |
| --- | --- | --- |
| 1 | HER2 (p.Y772_A775dup) | KRAS (p.G12D), TP53 (p.Y205H) |
| 2 | HER2 (p.Y772_A775dup) | BRAF (amlification), CDK6 (amlification), EGFR (amlification), HER2 (p.Y772_A775dup,amplification), MET (amlification), BIRC5 (amlification), EZH2 (amlification), GLI3 (amlification), SMO (amlification) |
| 3 | CREBBP (p.Q1765Rfs*6), HER2(p.Y772_A775dup) | EGFR (amlification), HER2 (p.Y772_A775dup, amplification), NRG1 (amlification) |
| 4 | HER2 (p.Y772_A775dup) | ATM (p.L1498*) |
| 5 | HER2 (G776delinsVC) | None |
| 6 | ARID1A (p.Q512*), HER2 (p.Y772_A775dup), RB1 (p.T373Qfs*6) | ARID1A (p.Q512*), HER2 (p.Y772_A775dup), RB1 (p.T373Qfs*6) |
| 7 | AKT2 (amplification), CCNE1 (amplification), HER2 (p.G776delinsVC), NKX2-1 (amplification), RB1 (p.Q395Nfs*6), TP53 (c.783-2A>G) | HER2 (p.G776delinsVC), RB1 (p.Q395Nfs*6), TP53 (c.783-2A>G) |
| 8 | HER2 (p.Y772_A775dup) | EP300 (p.E1715*), HER2 (p.Y772_A775dup) |
| 9 | DICER1 (p.S1344*), HER2 (p.Y772_A775dup), NFKBIA (amplification), NKX2-1 (amplification), TP53 (c.673-2A>C) | HER2 (p.Y772_A775dup), TP53 (c.673-2A>C) |
| 10 | DDR2 (amplification), HER2 (p.Y772_A775dup, amplification), NTRK1 (amplification), RIT1 (amplification), TP53 (p.Y220C), ZNF217 (amplification) | APC (p.K1878Rfs*4), HER2 (p.Y772_A775dup, amplification), FAM135B (amlification), GNAS (amlification), PREX2 (amlification), PTK2 (amlification), RICTOR (amlification), TP53 (p.Y220C), ZNF217 (amlification) |
| 11 | BRCA2 (p.P2802Lfs*19), HER2 (p.Y772_A775dup), PIK3CA (p.R108H), TP53 (p.Q317*) | HER2 (p.Y772_A775dup), PIK3CA (p.R108H), TP53 (p.Q317*) |
| 12 | HER2 (p.Y772_A775dup), LRP1B (p.Y3617*), TP53 (p.C176F) | HER2 (p.Y772_A775dup), FAT1 (p.N1594Mfs*18), NKX2-1 (amlification), TP53 (p.C176F) |

*, stop codon; fs, frameshift


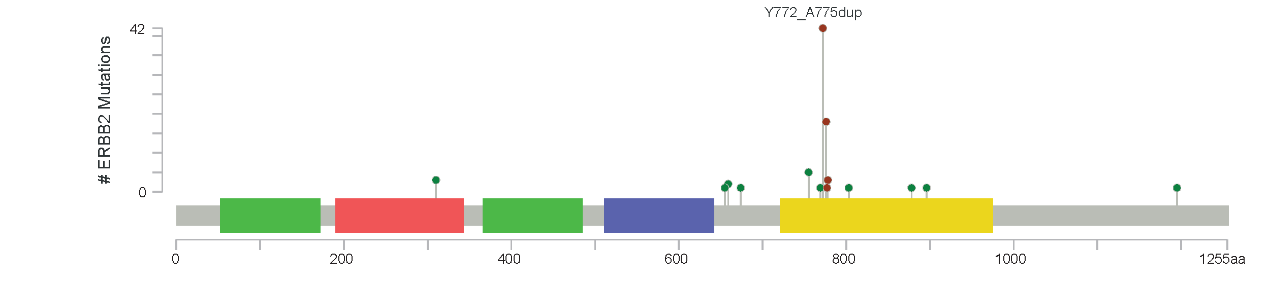


**Supplemental figure 1. *HER2* mutational map at baseline.** Green: receptor L domain; red: furin-like cysteine rich region; blue: growth factor receptor domain IV; yellow: protein tyrosine kinase.


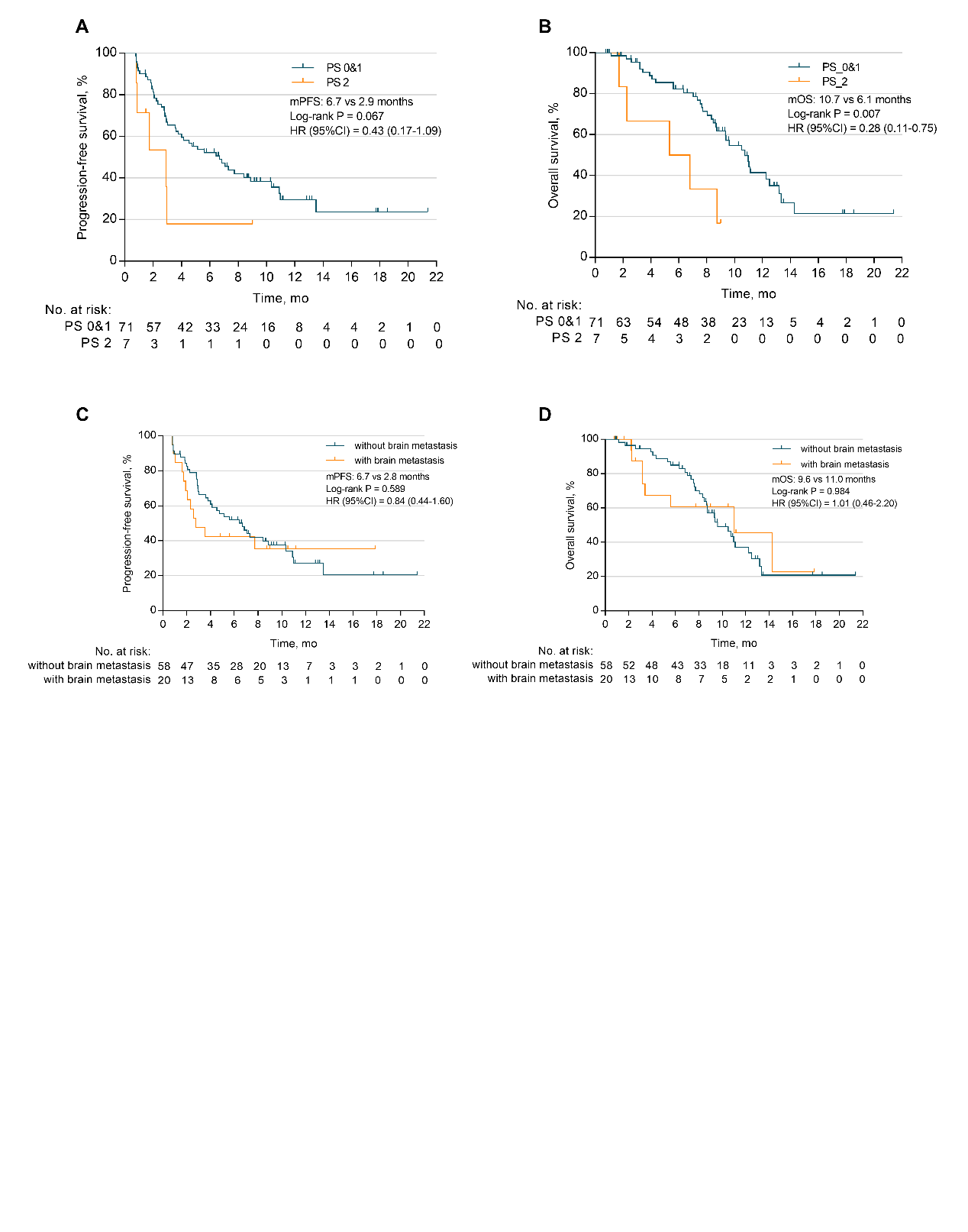


**Supplemental figure 2. Survival curves of pyrotinib treated *HER2*-mutated NSCLC patients according to baseline characteristics.** (A, B) progression-free survival (PFS) and overall survival (OS) according to the ECOG performance status. PS represents ECOG performance score. (C, D) PFS and OS of pyrotinib treated patients with or without brain metastasis. mPFS, median progression-free survival; mOS, median overall survival; HR, hazard ratio; 95%CI, 95% confidence interval.


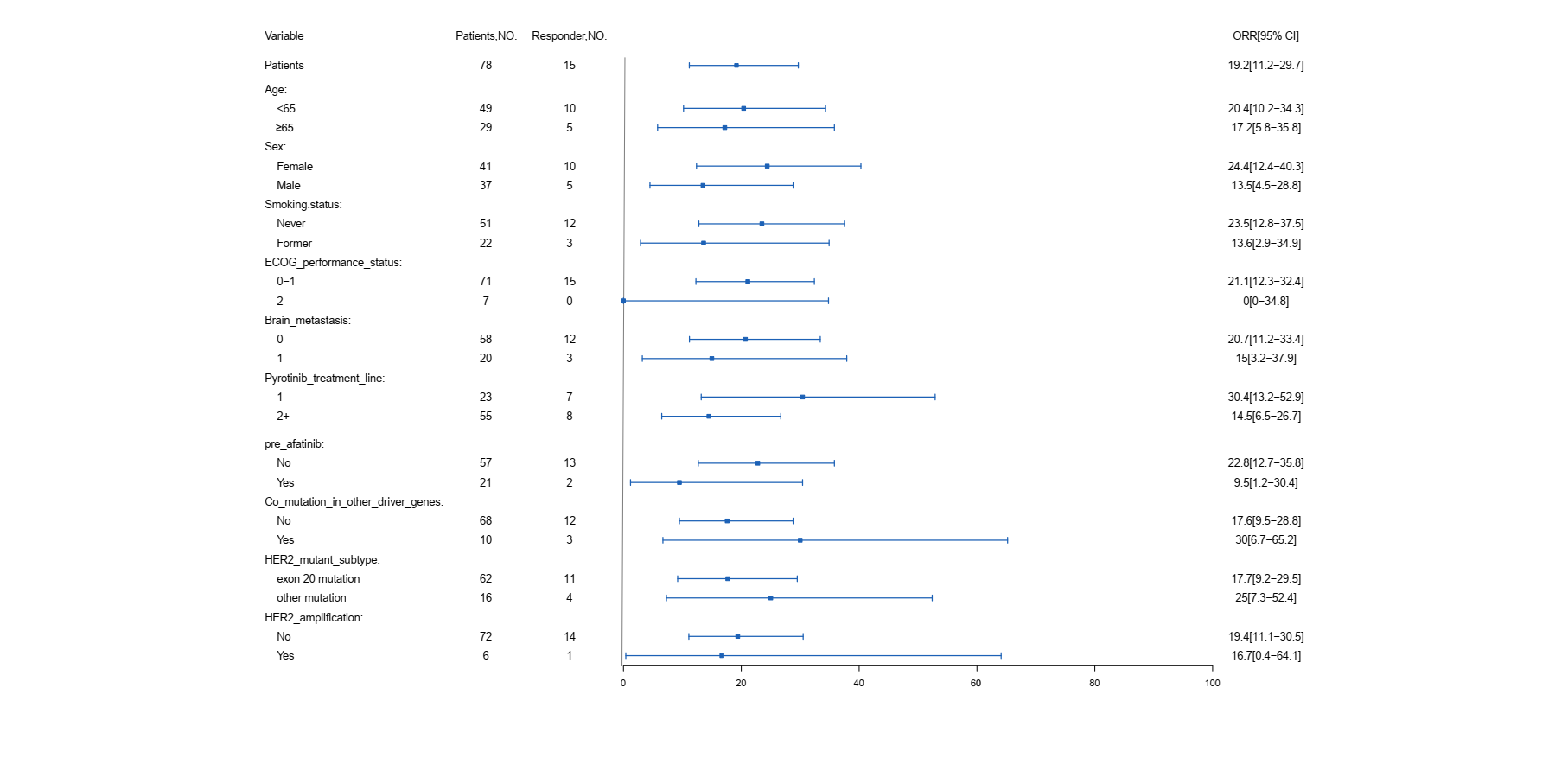


**Supplemental figure 3. Objective response rate in pre-specific subgroups.**


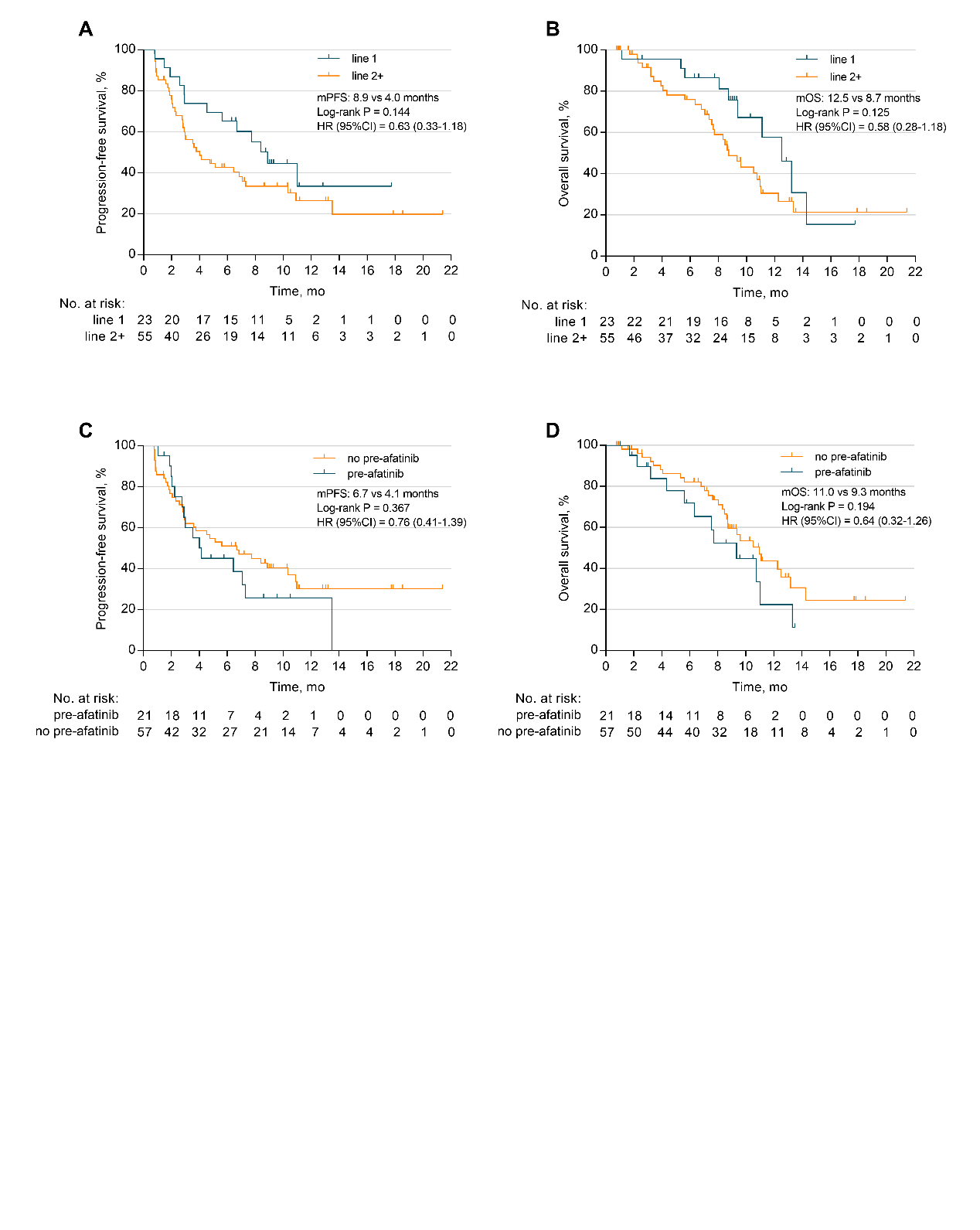


**Supplemental figure 4. Survival curves of NSCLC patients treated with pyrotinib according to previous treatment.** (A, B) progression-free survival (PFS) and overall survival (OS) according to the treatment lines of pyrotinib. (C, D) PFS and OS of patients according to the prior exposures to afatinib. mPFS, median progression-free survival; mOS, median overall survival; HR, hazard ratio; 95%CI, 95% confidence interval.


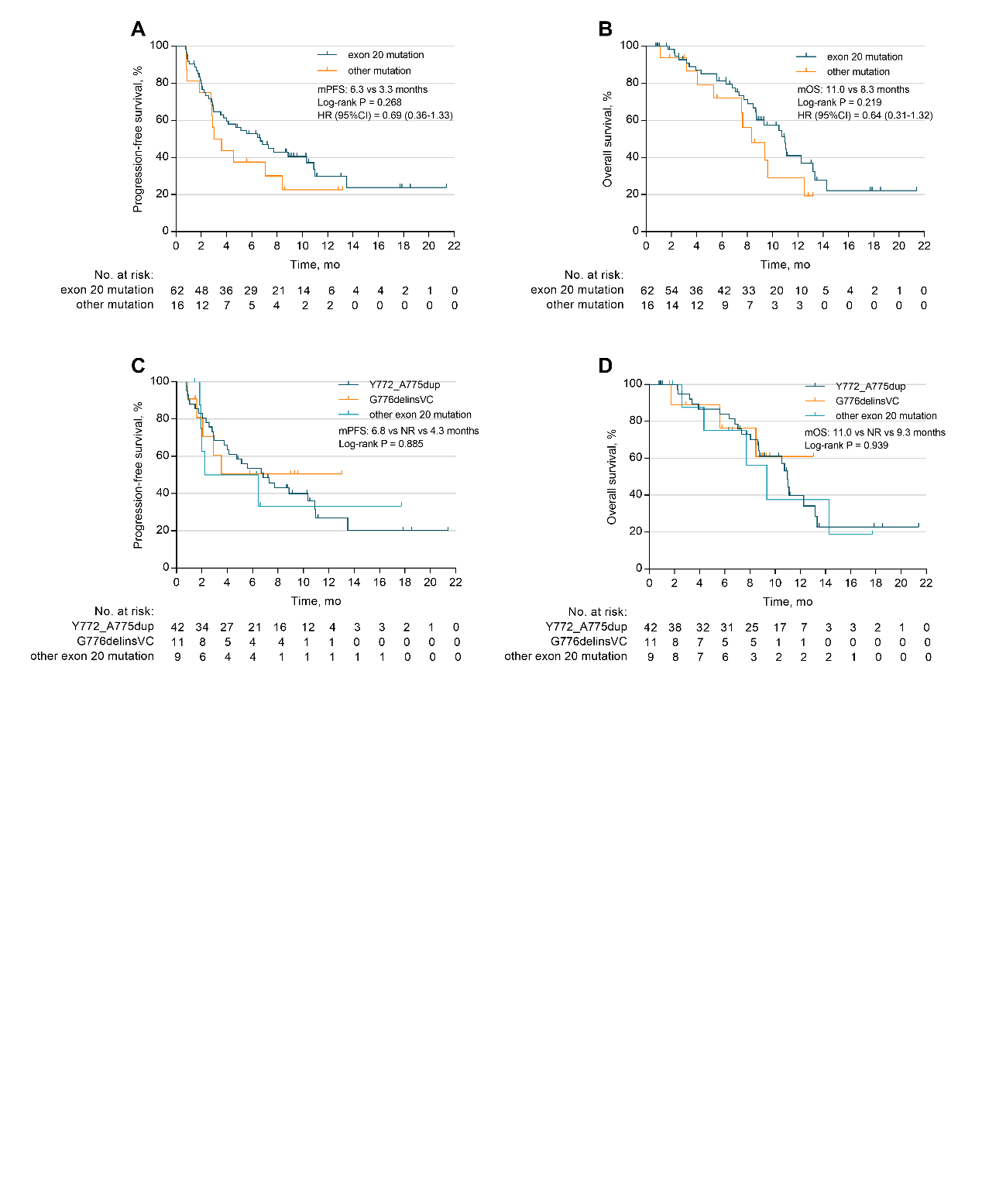


**Supplemental figure 5. Survival curves of pyrotinib treated NSCLC patients with different *HER2* mutation.** mPFS, median progression-free survival; mOS, median overall survival; HR, hazard ratio; 95%CI, 95% confidence interval.


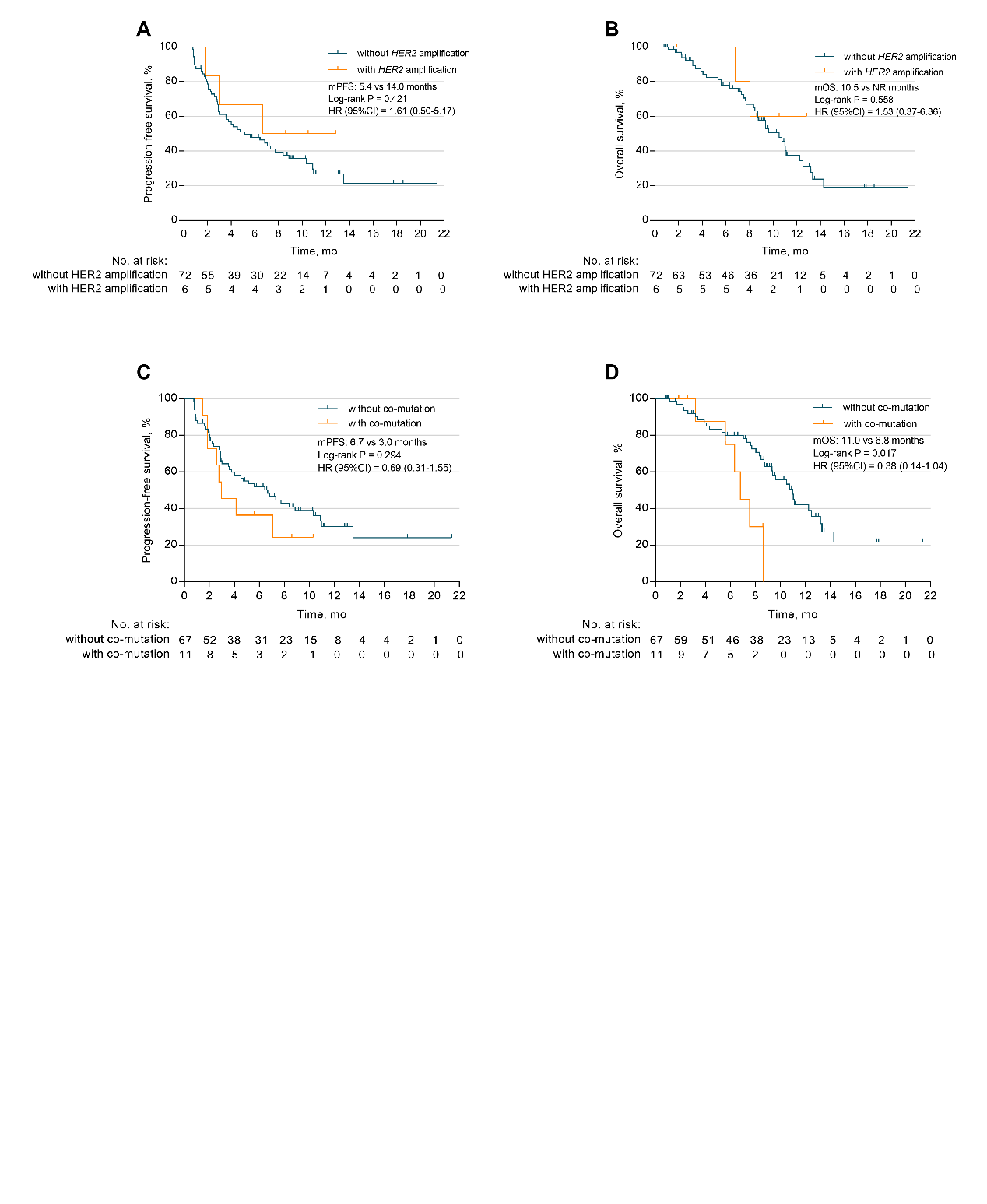


**Supplemental figure 6. Survival curves of pyrotinib treated *HER2*-mutated NSCLC patients according to molecular characteristics.** (A, B) progression-free survival and overall survival according to *HER2* amplification. (C, D) PFS and OS of pyrotinib treated patients according to the occurrence of co-mutations in other driver genes. mPFS, median progression-free survival; mOS, median overall survival; HR, hazard ratio; 95%CI, 95% confidence interval.


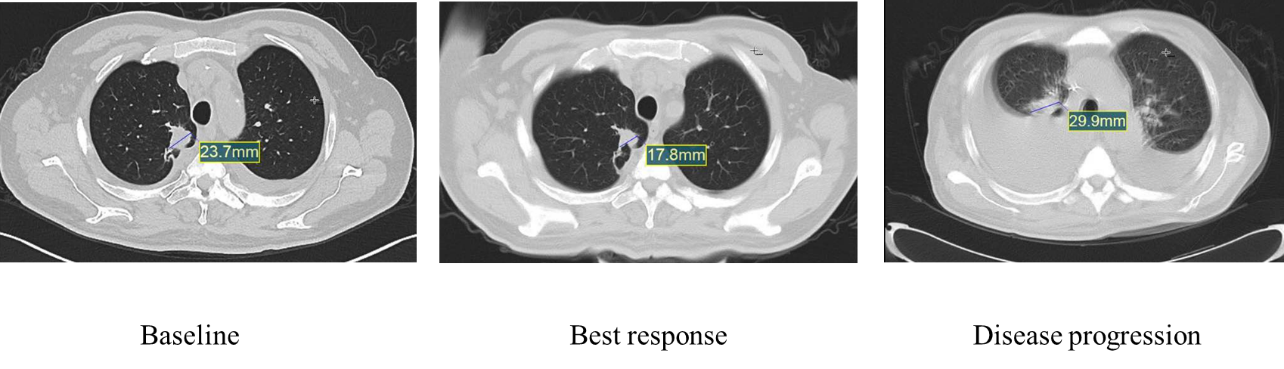


**Supplemental figure 7. Pyrotinib resistance in a patient with *HER2* and *EGFR* amplification.** CT scans were performed at baseline (2 weeks before starting pyrotinib), best response (2 months after starting pyrotinib) and disease progression (7 months after starting pyrotinib), respectively. Mm, millimeter.
